# Paracrine Molecules of Mesenchymal Stem Cells for Hematopoietic Stem Cell Niche

**DOI:** 10.1155/2011/353878

**Published:** 2011-09-22

**Authors:** Tian Li, Yaojiong Wu

**Affiliations:** Life Science Division, Graduate School at Shenzhen, Tsinghua University , Shenzhen L406A, China

## Abstract

Hematopoietic stem cells (HSCs) and mesenchymal stem cells (MSCs) are both adult stem cells residing in the bone marrow. MSCs interact with HSCs, they stimulate and enhance the proliferation of HSCs by secreting regulatory molecules and cytokines, providing a specialized microenvironment for controlling the process of hematopoiesis. In this paper we discuss how MSCs contribute to HSC niche, maintain the stemness and proliferation of HSCs, and support HSC transplantation.

## 1. Introduction

Hematopoietic stem cells (HSCs) are rare cells residing in the bone marrow (BM; 1 in 10^4^ to 1 in 10^8^ of BM nucleated cells), and they are progenitors that become progressively restricted to several or single lineages. These progenitors yield blood precursors devoted to unilineage differentiation and the production of mature blood cells, including red blood cells, megakaryocytes, myeloid cells (monocyte/macrophage and neutrophil), and lymphocytes [1, 2]. CD34 surface antigen (CD34^+^) is commonly used as a marker to identify and quantify the population of progenitor cells [3], according to which, sorting HSCs from BM, peripheral blood (PB), and umbilical cord (UC)/placenta blood is relatively simple and practical [2, 4–6]. Human HSCs are known to exhibit CD34^+^, Thy1^+^, CD38^lo/−^, Ckit^−/lo^, CD105^+^, and Lin^−^ phenotype. However, there is no general agreement on the association between any combination of these antigenic properties and function of stem cells [3, 6]. HSCs depend on their microenvironment, the niche, for regulating self-renewal and differentiation [7]. For instance, the disruption of BMP pathway can increase the numbers of osteoblasts and HSCs [8, 9], and the chemokine CXCL12 regulates the cyclical release and the migration of HSCs [10, 11]. Activation of *β*-catenin enforces HSCs enter cell cycle, thus leading to exhaustion of the long-term stem cell pool [12–14]. These findings suggest that signaling pathways and cellular interactions regulate the BM niche for HSCs. Besides, hypoxia regulate hematopoiesis in BM by maintaining important HSC functions and the interplay between HSCs and neighboring cells [15, 16].

Plating studies indicate that mesenchymal stem cells (MSCs) are present as a rare population of cells in the BM. They represent approximately 0.001% to 0.01% of the nucleated cells, about 10-fold less abundant than HSCs, but MSCs can be readily grown in culture [17]. Though predominantly residing in the BM, MSCs also present similar but not identical features in many other tissues such as blood, placenta, dental pulp, and adipose tissue. MSCs have the potential to differentiate into multiple phenotypes such as osteoblasts, chondrocytes, adipocytes, neural cells, and probably other cell lineages [18–21]. International Society for Cellular Therapy (ISCT) has provided the following minimum criteria for defining multipotent mesenchymal stromal cells as follows: plastic-adherent under standard culture conditions; express CD105, CD73, and CD90 and lack expression of CD45, CD34, CD14, or CD11b, CD79 or CD19 and HLA-DR, and must differentiate into osteoblasts, adipocytes, and chondroblasts *in vitro* [22].

BM has received the most attention because it carries MSCs as well as HSCs. Evidence indicates that MSCs are key component of the HSC niche in the BM where these two distinct stem cell populations arrange closely, ensuring hematopoietic and skeletal homeostasis [18]. MSCs interact with HSCs, secreting chemokines that contribute to HSC niche and support long-term growth of HSCs [23, 24]. MSCs can be cotransplanted with HSCs to improve their engraftment [25–27] (Table 1).

## 2. Mesenchymal Stem Cells Contribute to Hematopoietic Stem Cell Niche

The term “niche” for the specific HSC BM microenvironment was first coined in 1978, proposing that HSCs are in intimate contact with the bone, which was responsible for the apparently unlimited capacity of HSCs' proliferation and the inhibition of HSCs' maturation [38]. Niches exist within the BM which preserve specific aspects of hematopoiesis, such as HSC survival, self-renewal, and differentiation, supporting the maintenance of the blood system under normal and stressed conditions [39]. Research has made it increasingly clear that the stem cell niches provide a microenvironment which is important in protecting the self-renewing, undifferentiated state of their residents [40]. Three types of HSC niches have been hypothesized, defined according to the HSC uniformity [18, 41]. Two of these proposed niches are provided by cells directly descending from MSCs: the osteoblastic niche, where HSCs reside in close contact with endosteal cells [8], and the reticular stromal niche, where HSCs reside in close contact with stromal cells which are also known as mural cells or pericytes, the smooth muscle cells lining arteriolar side of the sinusoids [42]. The third proposed niche is the vascular/sinusoidal niche, where HSCs reside in direct contact with endothelial cells in the venous side of the sinusoids [43]. It is well known that HSC circulation involves HSCs leaving the BM, entering the vascular system (mobilization), and returning to the BM (homing) [44, 45]. The BM vascular structure provides a barrier between the hematopoietic compartment and the peripheral circulation. Most primitive HSCs remain physiologically quiescent within the BM niche; however, a portion of HSCs leave this resting pool and start the process of mobilization [39, 46–48]. 

Studies showed that both mouse and human osteoblast cell lines secreted a large number of cytokines that promote the proliferation of haematopoietic cells in culture, proving that cells involved in bone formation have stem-cell-supporting activity [49, 50]. MSCs reside in the bone cavity and are proposed to give rise to the majority of marrow stromal cell lineages, including chondrocytes, osteoblasts, and adipocytes, as suggested in numerous studies [48–50]. MSCs and HSCs form a structurally unique niche in the BM, which is regulated by local input from the surrounding microenvironment, and long-distance cues from hormones and the autonomic nervous system [51]. MSCs isolated from BM produce several growth factors and chemokines, such as CXCL12 (SDF-1), stem cell factor (SCF), Flt-3 ligand (FL), thrombopoietin (TPO), interleukin (IL)-6, IL-11, leukemia inhibitory factor (LIF), macrophage colony-stimulating factor (M-CSF), tumor necrosis factor- (TNF-) *α*, and transforming growth factor- (TGF-) *β*1 [28, 52–54]. HSCs are reduced in the BM after the depletion of MSCs, owing at least in part to mobilization towards extramedullary sites [51]. Loss of SCF from supporting cells or the receptor in HSCs leads to hematopoietic failure, indicating MSCs play an essential role in HSC niche function [36]. SCF and FL are implicated in maintaining HSC proliferation and self-renewal, regulating hematopoietic growth [28]. IL-3 or IL-6 combined with TPO signaling can influence HSC proliferation and differentiation [29, 34]. Besides, as mentioned previously, the chemokine CXCL12 interacts with its receptor CXCR4, regulates the cyclical release of HSCs, the migration of HSCs to the vascular niche from BM, and the homing of HSCs to the BM [10, 11, 29–32], and promotes adhesive interactions between HSCs and stromal cells [55]. In addition, CXCL12 chemokine signaling pathway contribute to the *ex vivo* expansion of HSCs [28]. Moreover, CXCL12 mediates angiogenic responses, promotes differentiation of CD34^+^ cells to endothelial progenitor cells, and appears to affect many other factors, including G-CSF, VEGF, and CXCL16 that relate to HSC mobilization and homing [33]. However, only *β*-catenin-activated MSCs but not naïve MSCs have stimulatory effect on HSC self-renewal *in vivo* [56].

## 3. The Effect of Mesenchymal Stem Cell on the Maintenance of Hematopoietic Stem Cells

Coculture of HSCs with MSCs might be an ideal method for maintaining the HSC pluripotency, because the growth or survival signals might be transferred to the HSC via adhesive molecules by modulating the cytokines and growth factor-dependent signals [57]. 5-aza-deoxycytidine (aza-D) and trichostatin A (TSA) have potent activity to maintain the stemness of HSCs, being candidate additives for HSCs *ex vivo* expansion, but they can also cause serious cell death [58, 59]. Koh et al. examined the effects of MSCs on the maintenance of CD34^+^ cells driven by aza-D and TSA in culture with the combined cytokines, and found that the total cell number of HSCs cultured with MSCs was higher in aza-D or TSA than in any culture conditions without MSCs, while most of HSCs cultured with cytokine treatment but without MSCs would lose their pluripotency and then differentiate, though they were induced to proliferate effectively [60]. It suggested that the co-culture of CD34^+^ cells with MSCs might not simply deliver the proliferation signals but also stemness and survival signals, and overlap the action of epigenetic regulators [57, 60].

## 4. Application of Bone Marrow Mesenchymal Stem Cells in Hematopoietic Stem Cell Transplantation

HSCs were primarily used in the treatment of patients with hematological malignancies. During the course of treatment, patients' cancerous cells are first destroyed by chemo/radiotherapy and then replaced with BM or PB/G-CSF transplant from a human leukocyte antigen- (HLA-) matched donor [61, 62]. In most cases, autologous HSCs are collected prior to the treatment and reinfused into the patients, but the patient's cancerous cells may be inadvertently collected and reinfused back into the patients along with HSCs [63]. Allogeneic marrow transplants have also been used in the treatment of hereditary blood disorders including aplastic anemia, *β*-thalassemia, Wiskott-Aldrich syndrome, and SCID, as well as inborn errors of metabolism disorders such as Hunter's syndrome and Hurler's syndrome [64–68]. One of the major challenges with HSC transplants is failure to engraft, which is mediated by donor T cells as a result of graft-versus-host disease (GVHD). Graft-versus-tumor effect of allogeneic HSC transplants may be a result of an immune reaction between donor cytotoxic T cells and patient's malignant cells [69]. MSCs are known to interact with HSCs and immune cells, and represent potential cellular therapy to enhance allogeneic hematopoietic engraftment and prevent GVHD [70–72]. Coculture of MSCs and HSCs could cause a significantly increase in CD34^+^ cells [73]. Aside from BM-derived MSCs, MSCs from adipose tissue can also be applied in hematopoietic engraftment, which would be an innovative supplement for cellular therapies [74, 75]. 

Cotransplantation studies in animal models as well as in humans showed that primary or culture-expanded MSCs promote the engraftment of HSCs. Cotransplantation of MSCs and cord blood or mobilized peripheral blood CD34^+^ cells resulted in a significantly higher level of engraftment than transplantation of CD34^+^ cells only [35, 37, 76–81]. This enhancement was greater after cotransplantation of GM-CSF and SCF-transfected MSCs, indicating that these growth factors relate to engraftment, though the mechanism of the enhancing effect is still unknown [35]. It is likely that the ability to promote engraftment is maintained along lineage differentiation [76]. Several lines of evidence suggest that MSCs produce several essential hematopoietic growth factors, adhesion molecules [28, 52–54], and extracellular matrix (ECM) proteins (such as VCAM1, E-selectin, collagen I, and fibronectin) that are known to play an important role in HSC homing [35, 37]. Selective adhesion of progenitors and cytokines to ECM components or stromal cells then result in the colocalization of progenitors at a specific stage of differentiation with a specific array of cytokines in so-termed niches [77]. This provides a level of growth and differentiation regulation [37]. Although it would mean exposure to allogeneic donor antigens, allogeneic MSCs can provide equal enhancement of engraftment as autologous cells. Cotransplanted MSCs shift the differentiation pattern from a lymphoid to a myeloid predominance and enhance megakaryocytic engraftment [78]. The cotransplantation of HSCs and MSCs enhanced engraftment as the dose of MSCs increased whereas an excessive dose of MSCs might decrease engraftment efficiency [79]. Besides, human allogeneic MSC layers in a serum-free culture system enabled the *ex vivo* expansion/maintenance of human HSCs [80], which indicates that MSCs may be used as a universal and reproducible stromal feeder layer to efficiently expand and maintain human BM HSCs *ex vivo* [81]. 

**Figure 1:**
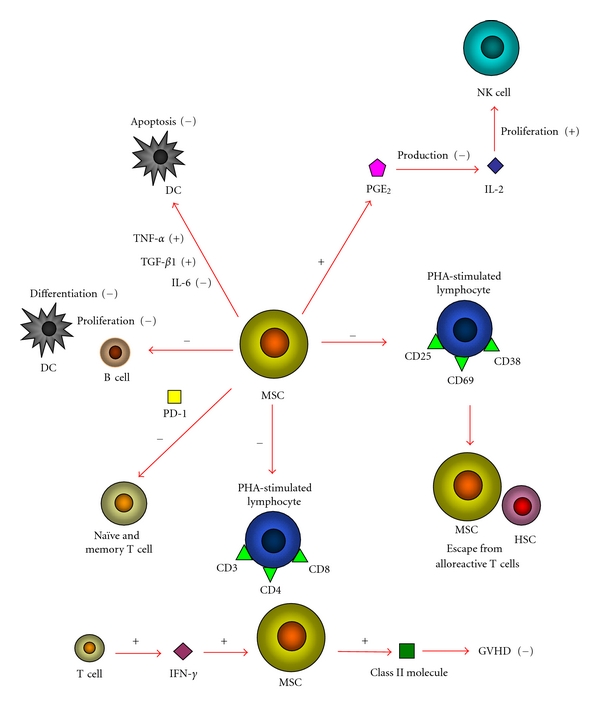
MSCs interact with immune cells, representing potential cellular therapy to enhance allogeneic hematopoietic engraftment and prevent GVHD. MSCs reduced the expression of activation markers CD25, CD38 and CD69 on PHA-stimulated lymphocytes, making allogeneic HSCs and MSCs escape from recognition of alloreactive T-cells. MSCs suppressed the proliferation of PHA-stimulated CD3+, CD4+ and CD8+ lymphocytes. MSCs inhibit naïve and memory T-cell responses to their cognate antigens by the engagement of the inhibitory molecule PD-1. MSCs inhibit the proliferation of B-cells and the differentiation of mature DCs from HSCs. MSCs induce DC apoptosis by downregulate TNF-*α* and TGF-*β*1 levels and upregulated IL-6 levels. MSCs inhibit the IL-2-induced proliferation of NK cells by producing PGE2. IFN-*γ* can stimulate MSCs to exhibit induction of class II molecule expression to prevent GVHD.

MSCs produce a microenvironment supporting hematopoiesis and may contribute to immune tolerance because of low immunogenicity and the suppressive effect of alloreactivity [75, 82]. MSCs had a potent immunosuppressive effect *in vivo* after allogeneic stem-cell transplantations [26]. The CXCL12-*α* secreted by MSCs could reduce the production of a variety of inflammatory cytokines and chemokines, including IL-13, IL-3 R*β*, IL-4, IL-5, IL-9, IL-10, L-selectin, MIP-3*α*/*β*, TCA3/CCL1, TNF-a, IL-1*β*, lymphotactin/CXCL1, L-selectin, leptin receptor, eotaxin-2, CTACK/CCL27, CRG-2/CXCL10, and CD30L [33]. In allogeneic transplantation, the simultaneous infusion of MSCs may promote hematopoietic engraftment across the major histocompatibility complex (MHC) barrier and decrease the incidence of GVHD, even though the exact mechanisms have not been clarified [83–85]. MSCs are lack of MHC class II and most of classical costimulatory molecules [86, 87]. Moreover, MSCs directly inhibit the expansion and activation of alloreactive Tlymphocytes and this T cell-suppressive effect may have important therapeutic implications in preventing or treating acute and chronic GVHD [70]. MSCs can significantly reduced the expression of activation markers CD25 (interleukn-2 receptor), CD38, and CD69 on phytohaemagglutinin- (PHA-) stimulated lymphocytes, making allogeneic HSCs and MSCs escape from recognition by alloreactive T-cells, because the expression of CD25 (IL-2 receptor), CD38 and CD69 was unchanged. Besides, MSC suppressed the proliferation of PHA-stimulated CD3^+^, CD4^+^, and CD8^+^ lymphocytes [87–89]. However, MSCs inhibit naïve and memory T-cell responses to their cognate antigens by the engagement of the inhibitory molecule PD-1 while the expression of MHC molecules and the presence in culture of antigen-presenting cells (APCs) or CD4^+^/CD25^+^ regulatory T cells were not required for MSCs to inhibit preferentially [87–91]. MSCs can regulate B-cell functions including migration, proliferation, and immunoglobulin(Ig) synthesis. For example, MSCs inhibit the proliferation of B-cells by arresting them at G0/G1 phase of the cell cycle, and the production of IgM, IgA, and IgG of B-cells [88, 92]. Dendritic cells (DCs) play an important role in supporting antigen-specific CD4^+^ T-cell proliferation and modulating diverse T-cell responses including GVHD [93]. MSCs can inhibit the differentiation of mature DCs from HSCs by arresting them at the precursor stage, interfere with DC antigen presentation, prevent DC migration ability, and induce DC apoptosis by downregulate TNF-*α* and TGF-*β*1 levels and upregulated IL-6 levels [93–95]. IFN-*γ*, which is produced by donor T-cells in response to antigen recognition, displays natural cytolytic activity against the cells missing markers of self-MHC class I, serves as an initiating stimulus for MSC immunosuppressive activity *in vivo *[88]. This indicates that the exposure to concentrated amounts of IFN-*γ* of MSCs can stimulate MSCs to exhibit induction of class II molecule expression, to prevent GVHD and provide the basis for a new potential strategy in prevention of GVHD [87–89, 96]. There is also evidences that MSCs can inhibit the IL-2-induced proliferation of natural killer (NK) cells by producing prostaglandin E_2_ (PGE_2_), a product of arachidonic acid metabolism that acts as a powerful immune suppressant, and inhibits T-cell mitogenesis and IL-2 production [88, 97, 98] (Figure 1).

## 5. Conclusion

Lines of evidence have indicated that MSCs are capable of supporting the expansion and differentiation of HSCs and enhancing hematopoietic engraftment in the past two decades, but the exact mechanisms by how MSCs support HSCs are still unclear. MSCs may affect HSCs by producing growth factors and chemokines that take parts in signaling pathways regulating HSCs. Meanwhile, HSCs interact with MSCs though this has been less understood. MSCs can home to injured tissues when coinfused with HSCs [99]. A better understanding of the interaction between MSCs and HSCs will substantially ultimately help develop novel therapies for hematopoietic diseases.

## Figures and Tables

**Table 1:** The cytokines secreted by MSCs that regulate HSCs.

Cytokines	Function	References
CXCL12	Regulate the adhesion, expansion, migration, and homing of HSCs	[10, 11, 28–32]
(SDF-1)	Reduce the production of inflammatory cytokines and chemokines	[33]

FL	Maintain HSC proliferation and self-renewal, regulate hematopoietic growth	[28]

IL-6, TPO	Influence HSC proliferation and differentiation	[29, 34]

GM-CSF	Regulate HSC engraftment	[35]

SCF	Maintain HSC proliferation and self-renewal	
	Regulate hematopoietic growth	[28, 36]
	Regulate HSC engraftment	[35]

VCAM1, E-selectin, collagen I, fibronectin	Regulate HSC homing and adhesion	[35, 37]
